# Supplementary material for: Causal interplay between lactose intolerance and gut microbiota: a combined bidirectional Mendelian randomization and in vivo validation study
Source: Front Nutr. 2026 Jun 1;13:1803337. doi: 10.3389/fnut.2026.1803337 (PMC13265576; doi:10.3389/fnut.2026.1803337)
Supplement: Supplementary file 1 [file Data_Sheet_1.zip › supplementary materials/Forward/leave one out cross validation/ebi-a-GCST90027553.finngen_R12_E4_LACTONAS.pdf]

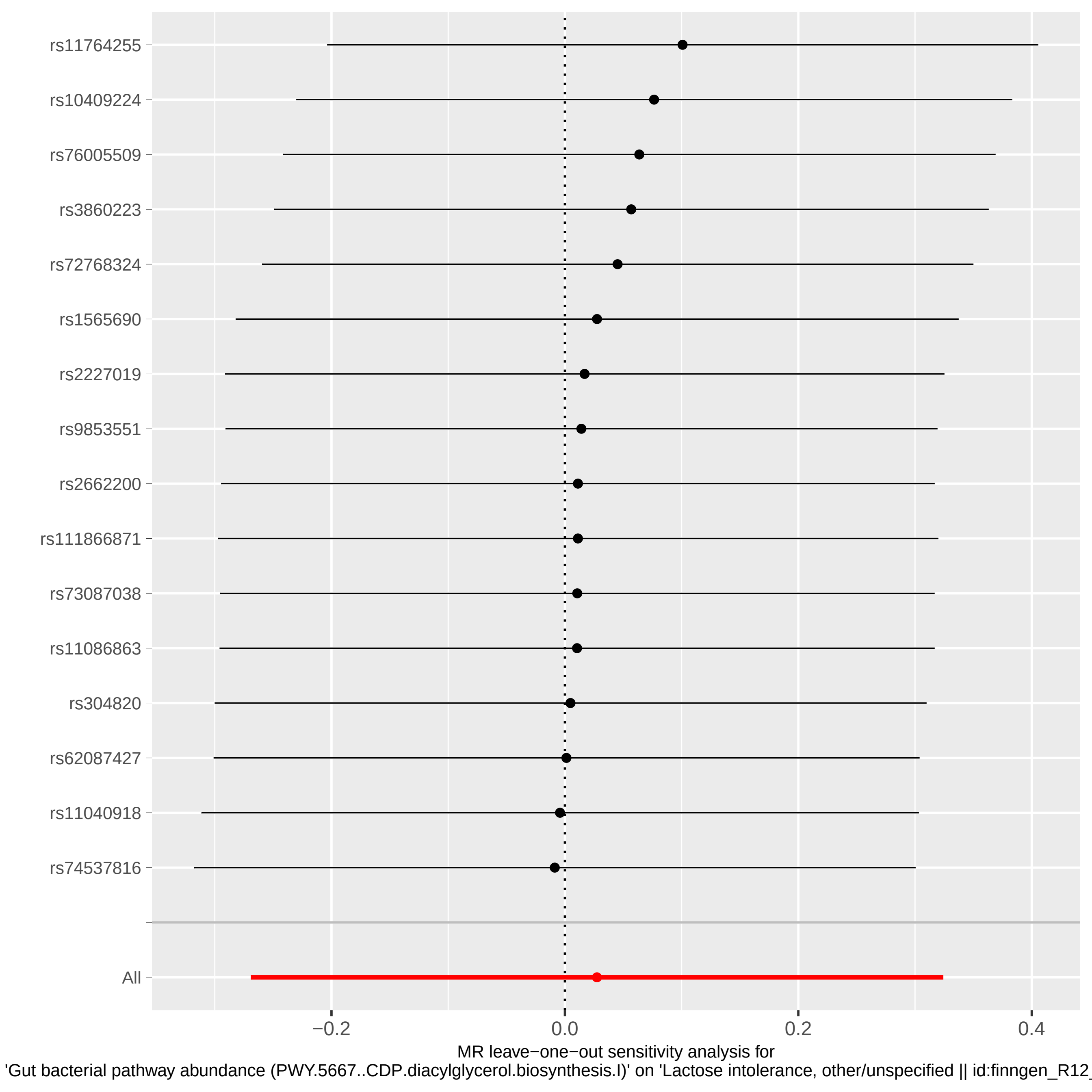
rs11764255
rs10409224
rs76005509
rs3860223
rs72768324
rs1565690
rs2227019
rs9853551
rs2662200
rs111866871
rs73087038
rs11086863
rs304820
rs62087427
rs11040918
rs74537816
All
−0.2
0.0
0.2
0.4
MR leave−one−out sensitivity analysis for
'Gut bacterial pathway abundance (PWY.5667..CDP.diacylglycerol.biosynthesis.I)' on 'Lactose intolerance, other/unspecified || id:finngen_R12_
